# Regulation of Cell-Signaling Pathways by Berbamine in Different Cancers

**DOI:** 10.3390/ijms23052758

**Published:** 2022-03-02

**Authors:** Ammad Ahmad Farooqi, Ru Wen, Rukset Attar, Simona Taverna, Ghazala Butt, Baojun Xu

**Affiliations:** 1 Department of Molecular Oncology, Institute of Biomedical and Genetic Engineering (IBGE), Islamabad 44000, Pakistan; farooqiammadahmad@gmail.com; 2 Department of Chemistry, University of Georgia, Athens, GA 30602, USA; r.wen3344@gmail.com; 3 Department of Obstetrics and Gynecology, Yeditepe University, Istanbul 34755, Turkey; ruksetattar@hotmail.com; 4 Institute for Biomedical Research and Innovation, National Research Council of Italy, 90146 Palermo, Italy; simona.taverna@cnr.it; 5 Institute of Translational Pharmacology (IFT-CNR), National Research Council of Italy, 90146 Palermo, Italy; 6 Institute of Botany, University of the Punjab, Lahore 54590, Pakistan; ghazala.botany@pu.edu.pk; 7 Food Science and Technology Program, BNU-HKBU United International College, Zhuhai 519087, China

**Keywords:** cancers, berbamine, cell signaling pathways, natural products, TGF/SMAD, delivery

## Abstract

Natural product research is a cornerstone of the architectural framework of clinical medicine. Berbamine is a natural, potent, pharmacologically active biomolecule isolated from *Berberis amurensis*. Berbamine has been shown to modulate different oncogenic cell-signaling pathways in different cancers. In this review, we comprehensively analyze how berbamine modulates deregulated pathways (JAK/STAT, CAMKII/c-Myc) in various cancers. We systematically analyze how berbamine induces activation of the TGF/SMAD pathway for the effective inhibition of cancer progression. We also summarize different nanotechnological strategies currently being used for proficient delivery of berbamine to the target sites. Berbamine has also been reported to demonstrate potent anti-cancer and anti-metastatic effects in tumor-bearing mice. The regulation of non-coding RNAs by berbamine is insufficiently studied, and future studies must converge on the identification of target non-coding RNAs. A better understanding of the regulatory role of berbamine in the modulation of non-coding RNAs and cell-signaling pathways will be advantageous in the effective translation of laboratory findings to clinically effective therapeutics.

## 1. Introduction

The deregulation of cell-signaling pathways is a central area of research in molecular oncology, and scientists have developed a nearly complete resolution of the multiple signaling cascades in different cancers. JAK/STAT and CAMKII/c-Myc are central drivers of carcinogenesis and metastasis. Moreover, TGF/SMAD has been shown to play a significant role in the regulation of carcinogenesis. Additionally, autophagy and apoptosis have fundamental roles in molecular oncology.

Nature has evolved over billions of years to generate an extra-ordinarily diverse array of natural products of wider structural variations and incredible complexities. Such widely heralded landmark discoveries as etoposide, teniposide, vinblastine and vincristine serve as monuments to the contributions of medicinal chemistry research over the decades.

Berbamine is a natural, potent, pharmacologically active biomolecule isolated from *Berberis amurensis*. Berbamine has a molecular weight of 608.7. There are some good reviews that have generally explored the pharmacological properties of berbamine [1,2,3]. However, a comprehensive overview of the regulation of signaling pathways by berbamine in carcinogenesis and metastasis has not yet been explored, which can underscore and identify the existing knowledge gaps in this field. We browsed PUBMED, using the keywords berbamine, cancer and metastasis. 

In this mini-review, we comprehensively analyze how berbamine modulates deregulated pathways (JAK/STAT, CAMKII/c-Myc) in various cancers. We have also systematically analyzed how berbamine induces the activation of the TGF/SMAD pathway for the effective inhibition of carcinogenesis.

## 2. Search Strategy

The search was conducted in PubMed, limited to articles published between 1 January 2000, and 31 December 2020. Research papers and review articles were included in the evaluation. Only articles published in the English language were considered. Relevant articles were searched as follows: (“Berbamine” OR “*Berberis amurensis*” AND “*”), where “*” stands for: (i) “cancer” OR “tumor” OR “tumour”; (ii) “JAK/STAT signaling” OR “JAK/STAT”; (iii) “TGF/SMAD Pathway” OR “TGF/SMAD” (iv) “Ca^2+^/calmodulin-dependent protein kinase II” OR “CAMKII”; (v) “Autophagy” OR “autophagy inhibitor”; (vi) “Nontechnology” OR “Nontechnological Approaches”; (vii) “Delivery”; (viii) cancer chemopreventive OR chemotherapy; (ix) “microRNA” OR “miR” OR “miRNA”. Articles were then sorted by relevance and screened for suitability to the scope of the review.

## 3. Regulation of JAK/STAT Signaling

JAK/STAT signaling has been shown to play a central role in different cellular processes [4,5]. Different types of cells in the tumor microenvironment secrete chemokines, leading to the activation of the JAK/STAT pathway in tumor cells and tumor-infiltrating immune cells, which can fuel proliferation, invasiveness and metastases. Increasing evidence clearly indicates that STAT proteins regulate many pathways important in tumorigenesis, including cell survival, tumor angiogenesis, loss of apoptosis, tumor cell invasion and metastasis.

Berbamine worked synergistically with sorafenib and combinatorically reduced basal and interleukin-6-dependent STAT3 activation in HCC cells [6]. Furthermore, berbamine and sorafenib combinatorically suppressed STAT3 phosphorylation at the 705th tyrosine residue, but STAT3 knockdown led to an abrogation of the sensitization effects of berbamine [6].

CAMKIIγ activated the ERK/STAT3 signaling pathway in multiple myeloma cells [7]. However, CAMKIIγ knockdown reduced the levels of p-ERK and STAT3 in multiple myeloma cells. CAMKIIγ overexpression significantly promoted the tumor growth of multiple myeloma cells in xenografted mice. Intraperitoneally administered berbamine and its novel analogue, WBC158, exerted tumor growth-inhibitory effects in mice xenografted with CaMKIIγ-expressing U266 cells [7].

Berbamine enhanced the chemosensitivity of gefitinib against PANC-1 and MIA PaCa-2 cancer cells [8]. Berbamine and gefitinib synergistically induced apoptotic death in pancreatic cancer cells. Berbamine physically interacted with STAT3 and inhibited its activation [8].

**Figure 1:**
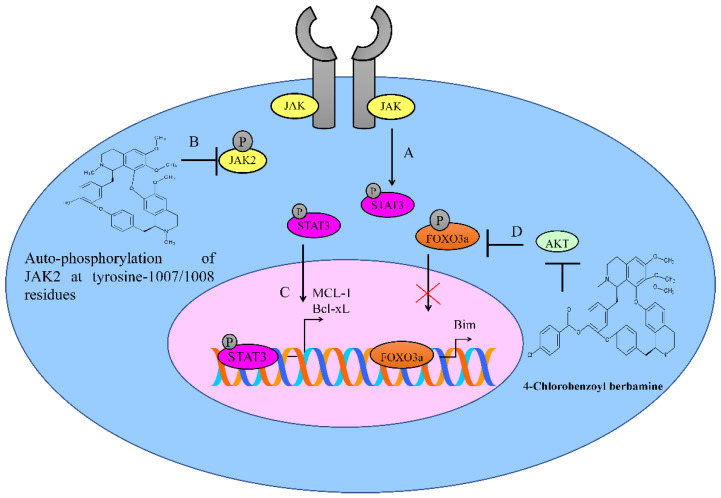
(**A**,**B**) JAK/STAT phosphorylation is involved in the regulation of different gene networks. Berbamine inhibits JAK2 auto-phosphorylation. (**C**) STAT3 transcriptionally upregulates MCL-1 and Bcl-xL. However, the inactivation of STAT3 leads to the repression of the STAT3-mediated expression of MCL-1 and Bcl-xL. (**D**) AKT inactivates FOXO3a and blocks the expression of pro-apoptotic Bim. Berbamine derivatives mediate inactivation of AKT, leading to an increase in FOXO3a-dependent upregulation of Bim.

The synthetic berbamine derivative directly inhibited the auto-phosphorylation kinase activity of JAK2 [9]. The auto-phosphorylation of JAK2 kinase at tyrosine-1007/1008 residues was potently inhibited in melanoma cells (Figure 1). Following the inhibition of JAK2 auto-phosphorylation, the berbamine derivative caused a blockade of constitutively activated downstream STAT3-signaling in melanoma cells. The berbamine derivative efficiently downregulated the expression level of the STAT3 target genes MCL-1 and Bcl-xL (Figure 1) [9].

Berbamine and radiation markedly reduced the levels of p-STAT3 in FaDu cells. Moreover, berbamine and radiation effectively induced a regression of the tumors in mice subcutaneously injected with FaDu cells [10]. 

4-Chlorobenzoyl berbamine, a novel berbamine derivative, inhibited the autocrine production of interleukin-6 and downregulated membrane interleukin-6 receptor expression [11]. The berbamine derivative inhibited the activation of STAT3 and AKT. AKT inactivated FOXO3a and blocked the expression of pro-apoptotic Bim. However, berbamine derivatives mediated the inactivation of AKT, which led to an increase in the FOXO3a-dependent upregulation of Bim in U266 cells (Figure 1) [11]. 

## 4. Regulation of TGF/SMAD Pathway

The TGF/SMAD signaling pathway has been extensively studied and is reportedly involved in the context-dependent regulation of cancer onset and progression. The SMAD protein family is functionally characterized into three sub-categories: R-SMAD (SMAD1, SMAD2, SMAD3, SMAD5 and SMAD8), I-SMAD (SMAD6 and SMAD7) and co-SMAD (SMAD4) [12]. Receptor-regulated SMADS (R-SMAD) had a C-terminal SSXS motif that was phosphorylated by type I receptors. Therefore, the phosphorylation of R-SMAD allowed its interaction with SMAD4. After the accumulation of the phosphorylated-R-SMAD/SMAD4 hetero-trimerically assembled complex into the nucleus, SMADs interacted with different transcriptional factors and co-regulators for the regulation of the expression of target genes [13,14,15,16].

Previous studies have shown that berbamine induced an increase in the expression levels of SMAD3 and p21 [17]. However, the expression levels of TGFβRI and TGFβRII did not change significantly. There was a marked increase in the levels of total SMAD3 and phosphorylated-SMAD3 in Berbamine-treated chronic myeloid leukemia cells [17]. Collectively, these findings suggest that berbamine exerts inhibitory effects on c-Myc and cyclin D1 through p-SMAD3. 

In another study, Berbamine was found to be more effective when used in combination against pancreatic cancer cells. Berbamine worked synergistically with gemcitabine and activated the TGFβ/SMAD pathway [18]. There was a decrease in the protein level of SMAD7, whereas there was an increase in TβRII levels. c-Myc and cyclin D1 were found to be reduced in BxPC-3 and PANC-1 cancer cells combinatorically treated with berbamine and gemcitabine [18].

Overall, these studies provide evidence that berbamine-induced activation of the TGF/SMAD pathway is essential for cell cycle arrest and the induction of apoptosis in cancer cells. Berbamine-mediated activation of the TGF/SMAD pathway has been partially uncovered in these studies. It has also been noted that berbamine inhibited negative regulators of TGF/SMAD signaling in order to allow nuclear SMADs to regulate the gene network. 

## 5. Targeting of Ca^2+^/Calmodulin-Dependent Protein Kinase II (CAMKII) by Berbamine in Different Cancers

Targeting c-Myc with small-molecule inhibitors remains challenging. c-Myc protein stability can be controlled by phosphorylation at two different sites with opposing functions. Phosphorylation at 62nd serine residue stabilized c-Myc, whereas phosphorylation at 58th threonine residue promoted the degradation of c-Myc. Ca^2+^/calmodulin-dependent protein kinase II (CAMKII), a multifunctional serine/threonine kinase, has been shown to stabilize the oncogenic c-Myc level [19]. Expectedly, levels of phosphorylated-c-Myc (62nd serine residue) and total c-Myc were noted to be reduced in CAMKIIγ knockdown cells, whereas levels of phosphorylated-c-Myc (62nd serine residue) and total c-Myc were found to be significantly enhanced in CAMKIIγ overexpressing T cell lymphoma cells [19].

An orally administered, bioactive small molecule analog of berbamine, tosyl chloride-berbamine (TCB), considerably reduced phosphorylated levels of CaMKIIγ [20]. TCB induced a regression of leukemia growth in an orthotopic B-ALL model using NSG (NOD/SCID/IL2Rγ-/-) mice injected with CaMKIIγ/Myc-expressing leukemia cells [20].

Berbamine had the ability to bind to the ATP-binding pocket of CaMKIIγ, inhibiting its phosphorylation and inducing apoptosis in leukemia stem cells [21]. 4-Chlorobenzoyl berbamine (CBBM), a Berbamine derivative, effectively enhanced the proteasome-dependent degradation of c-Myc in OCI-Ly3 cells [22].

Berbamine has been reported to block VEGF- and BDNF-regulated angiogenesis, mainly through the inactivation of VEGFR2- and TrkB-mediated transduction cascades [23]. Berbamine considerably reduced VEGF-dependent phosphorylation of VEGFR2, as well as that of TrkB by BDNF in HUVECs, resulting in the deactivation of downstream effectors, such as PKB/AKT, NF-κB and ERK1/2. Berbamine efficiently reduced BDNF and VEGF-mediated CaMKIIγ phosphorylation. Berbamine significantly suppressed tumor growth in chorioallantoic membrane tumor models implanted with U87MG cells [23]. 

Berbamine and one of its derivatives, BBD24, strongly inhibited CAMKII phosphorylation in Huh7 cells [24]. Overall, these studies helped us develop a sharper understanding of the instrumental role of the CAMKII/c-Myc-signaling axis in carcinogenesis. 

## 6. Regulation of Autophagy by Berbamine

Berbamine is a natural molecule from traditional Chinese medicine that is useful for the treatment of patients with inflammation and cancers such as leukaemia, lung, liver and breast cancer. Berbamine is administered to patients with leukopenia caused by conventional chemotherapy and/or radiotherapy. Several reports indicate that berbamine has a role in the modulation of deregulated pathways in cancers. Berbamine causes caspase-3-dependent apoptosis in leukaemia cells through surviving pathway activation [25]. Moreover, berbamine inhibits the cell growth and motility of highly metastatic breast cancer and lung cancer cells [26,27].

Recently, berbamine has been considered a novel autophagy inhibitor in breast and colon cancer cells. Autophagy is an essential catabolic process involved in many pathological conditions, including cancer, that can protect cells and organisms from stressors. The role of autophagy in cancer remains uncertain and has been reported as a pro- and anti-tumorigenic system [28]. In pre-malignant lesions, autophagy activation might prevent cancer development [29]. Conversely, in advanced cancers, autophagy induction can stimulate carcinogenesis, such as in melanoma, colorectal, pancreas and renal cancers, or suppress it, as in breast cancer [30,31,32,33,34,35]. In 1988, Ohsumi described, for the first time, autophagy mechanisms as a lysosomal degradation and cellular recycling pathway, evolutionarily conserved, that allows protein aggregates and damaged organelles to be eliminated through lysosomal degradation, thus maintaining cellular homeostasis [36]. Autophagy can be divided into three groups: macroautophagy, microautophagy and chaperon-mediated autophagy. Macroautophagy (we refer to macroautophagy as autophagy) is the best known of the three pathways. Autophagy mediates the sequestration and delivery of cytoplasmic material to lysosome for degradation. This process induces the phagophore formation caused by the extension of an isolated membrane, which fuses to convey cytoplasmic components into an autophagic double membrane vacuole, the autophagosomes; the organelles fused with lysosomes become autolysosomes, which degrade the materials contained within it. The autophagy process is modulated at transcriptional and post-translational levels, and the genes involved in autophagy are regulated by ATF4 at the transcriptional level. Cellular stresses induce MIT/TFE transcription factors and other inhibitors of mTOR, a negative regulator of MIT/TFE. In the autophagy process, the fusion of membranes is usually realized by soluble SNARE complexes (N-ethylmaleimide-sensitive factor attachment protein receptor). Recently, it has been described that the SNARE syntaxin 17 (STX17) contained in autophagosomes interacts with the cytoplasmatic SNARE SNAP29 and SNARE VAMP8 of the lysosomes, and these proteins cooperate in autophagosome-lysosome fusion. 

**Figure 2:**
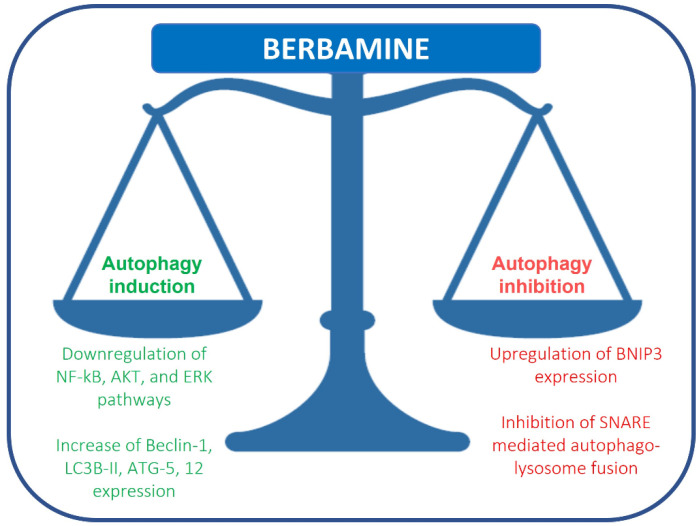
Regulation of autophagy by berbamine.

Fu et al. reported that berbamine causes the upregulation of BNIP3, inhibiting SNARE-mediated autophagy-lysosome fusion in breast cancer cells. Under hypoxia, BNIP3, a protein with homology to BCL2 in the BH3 domain, drives mitophagy in many different cell types. BNIP3 may play a role in autophagosome-lysosome fusion regulation. Berbamine induces the upregulation of BNIP3, which binds SNAP29 and inhibits the interaction between SNAP29 and VAMP8, which, in turn, causes a blockade of autophagosome-lysosome fusion and autophagosome increase (Figure 2) [37]. This study suggests that berbamine could be considered a potential autophagy inhibitor, which could be used in combination with chemotherapy in cancer management. Autophagic cell death can also be induced by RAS/RAF/MEK/ERK pathway activation [38]. Mou et al. reported that berbamine can exert anticancer effects on human colon cancer cells, inducing autophagy and apoptosis and inhibiting cell migration via the MEK/ERK pathway. Berbamine induces autophagic vesicles in colon cancer cells and an increase in Beclin-1, LC3B-II, ATG-5 and 12 expression [39]. 

Preliminary studies on berbamine indicate its role in cancer treatment. It is efficient because other natural compounds could be limited by reduced water solubility, low absorption rate in the bowels and rapid metabolism. The role of berbamine in autophagy is still unclear. Further studies are needed to elucidate the effects of this natural compound on autophagy regulation. 

## 7. Targeting of Protein Networks by Berbamine for Cancer Chemoprevention

Berbamine efficiently suppressed the migratory and invasive capacity of highly metastatic breast MDA-MB-231 cancer cells via the inhibition of pro-MMP2/MMP9 activation. It also reduced the phosphorylated levels of AKT and c-Met in MDA-MB-231 cancer cells [27]. 

Aspirin dose-dependently induced the phosphorylation of CREB and ATF1 [40]. Importantly, the treatment of HepG2 cells with AICAR (an AMPK agonist) also resulted in the phosphorylation of CREB and ATF1. However, knockdown of AMPKα1 abolished the phosphorylation of CREB/ATF1 by aspirin in Hep3B and HepG2 cells. PKA (Protein kinase A) is present downstream to AMPK and mediates the phosphorylation of CREB/ATF1 by aspirin. Accordingly, PKA inhibitors impaired the phosphorylation of CREB/ATF1 by aspirin in HepG2 and Hep3B cells. Soluble adenylyl cyclase (sAC) has an important role in cAMP synthesis. Aspirin did not induce phosphorylation of CREB/ATF1 in sAC knockdown cells. Aspirin caused marked reduction in the levels of CPS1 (carbamoyl phosphate synthetase I) in HepG2 and Hep3B cells. Similarly, AICAR (an AMPK agonist) inhibited CPS1 expression in HepG2 and Hep3B cells. AMPK knockdown abrogated the downregulation of CPS1 and upregulation of sAC by aspirin. CREB/ATF1 knockdown sensitized Hep3B and HepG2 cells to aspirin. CREB/ATF1 activation antagonized the anti-cancer effects of aspirin, and pharmacological targeting of CREB/ATF1 significantly enhanced the efficacy of aspirin against HCC cancer cells. Berbamine inhibited CREB/ATF1 phosphorylation and sensitized HCC cells to aspirin. Protein phosphatase-2A (PP2A) induced dephosphorylation of its substrates. However, CIP2A (a cellular inhibitor Of PP2A) negatively regulated PP2A. Berbamine reduced CIP2A levels in HCC cells and promoted PP2A-mediated abrogation of aspirin-induced phosphorylation of CREB/ATF1 [40].

Berbamine concentration-dependently inhibited the migratory and invasive potential of SMMC-7721 cells and increased expression of Cx32 in SMMC-7721 cells. However, after silencing Cx32, berbamine failed to inhibit cell invasion and metastasis [41].

## 8. Clues about Regulation of microRNAs

The novel berbamine derivative time- and dose-dependently induced apoptosis in cancer stem-like cells (CSCs) of human glioblastoma. The berbamine derivative induced an upregulation of microRNA-4284 of more than fourfold in GBM stem-like cells [42]. The use of antisense oligonucleotide against miR-4284 partially blocked the anticancer effects of the berbamine derivative on GBM stem-like cells. Structural studies have shown that dimeric AP-1 transcriptional factor complexes triggered cellular mechanisms via regulation of the target gene network in response to positive and negative stimuli. The novel berbamine derivative time- and dose-dependently induced apoptosis in cancer stem-like cells (CSCs) of human glioblastoma. The berbamine derivative increased the phosphorylation of JNK isoforms in the neurosphere cultures. Additionally, the berbamine derivative increased the phosphorylation of c-Jun and enhanced protein levels of total c-Fos [42]. Collectively, these findings suggest that the berbamine derivative activated a JNK-c-Jun/AP-1 transduction cascade for the induction of apoptosis. 

However, the role of miR-4284 needs to be tested more effectively in various cancers. Certain hints that point towards the oncogenic role of miR-4284 in gastric cancer have emerged. miR-4284 directly targeted TET1 (ten-eleven translocation 1) and promoted the tumor growth, migration and invasion of gastric cancer cells [43]. Therefore, miR-4284 has been shown to behave as a double-edged sword in different cancers. 

## 9. Nontechnological Approaches for Delivery of Berbamine in Different Cancers

Nanotechnology is widely applied in delivery systems to effectively improve the therapeutic index and reduce side effects in a lower drug load context than the native compound. Various materials can be made to nanoscale and used as drug carriers in many applications. Polymeric nanoparticles are one such delivery system due to their biocompatibility, biodegradability, tunable pharmacokinetics and circulation. Sulfobutylether-*β*-cyclodextrin/chitosan nanoparticles are developed to enhance oral bioavailability and improve small intestinal absorption [44]. Sulfobutylether-β-cyclodextrin/chitosan nanoparticles are able to co-load docetaxel (DTX) and berbamine using an ionic gelation method. These DTX-berbamine nanoparticles demonstrated better in vitro cancer cell death and apoptosis, as well as an improved pharmacokinetic profile, compared to free compound formulations in MCF-7 cells. Further analysis showed that DTX/berbamine nanoparticle inhibited surviving mRNA expression, suggesting NF-kB pathway involvement [45]. Further related mechanistic studies are necessary to confirm this pathway. However, there are no in vivo studies demonstrating tumor suppression and oral bioavailability.

In another study, poly(caprolactone) (PCL) was conjugated with methoxy poly (ethylene glycol) (mPE) as copolymers to utilize the hydrophobic portion of PCL for drug encapsulation and the hydrophilic part of mPE for drug delivery providing suitable aqueous solubility. The amphilic mPE-PCL block copolymers were self-assembled into nanospheres with a hydrophilic outer shell made of mPE and a hydrophobic inner core. The mPE-PCL copolymer is reported to construct polymeric nanoparticles co-delivering paclitaxel (PTX) and berbamine. PTX and berbamine were able to enter the cells via the nanoparticles, as confirmed by fluorescence caused by coumarin-6. The berbamine-PTX-loaded mPE-PCL nanoparticles demonstrated effective anti-cancer properties in human gastric cancer BGC823 cells in vitro, as well as superior tumor suppression, compared with free drugs in vivo. The paclitaxel/berbamine-loaded nanoparticles showed the best tumor suppression compared with free PTX, berbamine or paclitaxel/berbamine compound [46]. However, it is not clear where the berbamine ended up, and mechanistic pathways are not mentioned. Furthermore, more extensive research is required on other polymeric structures, such as FDA-approved poly(lactic-co-glycolic acid) (PLGA) and polyethylene glycol (PEG), in order to achieve subcellular localization for targeting therapy. PLGA-PEG-TPP might be able to deliver the compound into mitochondria by taking advantage of mitochondrial membrane potential. PLGA-PEG and PLGA-PEG-TPP nanoparticles could be promising delivery systems for berbamine to enhance its therapeutic intervention.

Lipid nanoparticles are alternative, rapidly developing carriers within the nanotechnology field in drug delivery due to their unique size-dependent properties and biodegradability. Parhi et al. reported that berbamine-loaded lipid nanoparticles with a diameter of 75 m showed effective anti-metastatic and anti-tumorigenic properties both in vitro and in vivo. The berbamine nanoparticles were made from a two-step process, in which berbamine was first incorporated into the fluid phase of glyceryl mono-oleate (GMO) and further emulsified with α-tocopherol poly (ethylene glycol) 1000 succinate (TPGS). The 6-coumarin-loaded nanoparticle showed that GMO-TPGS lipid nanoparticles were able to access A549 and MDA-MB-231 cells. The berbamine-GMO-TPGS nanoparticles showed superior cellular toxicity, as well as an inhibition of migration and invasion in metastatic breast cancer MDA-MB-231, lung cancer A549 and melanoma B16F10 cell lines when compared to native berbamine. An in vivo mouse study with a melanoma B16F10 subcutaneous tumor model demonstrated that berbamine-GMO-TPGS nanoparticles effectively suppressed primary tumor growth when compared with free berbamine. Berbamine-GMO-TPGS nanoparticles inhibited lung metastasis after 45 days of cell injection through the tail vein in the C57BL/6 mouse model [47]. 

## 10. Berbamine Mediated Cancer Chemopreventive Effects in Tumor-Bearing Mice

Berbamine effectively inhibited cell survival pathways. The PI3K/AKT-signaling axis was reported to be reduced [48]. The inhibition of p53-MDM2 interactions is a promising strategy for triggering the activation of p53. MDM2 not only exerts inhibitory effects on p53 transcriptional activity, but also promotes its nuclear export and subsequent degradation. Therefore, the disassembly of p53-MDM2 by certain synthetic or natural products potently enhances p53-mediated apoptosis in p53-positive cancer cells. MDM2-mediated inhibition of p53 was also found to be relieved in berbamine-treated lung cancer cells. Tumors that developed from A549 cancer cells were noted to be smaller in size in the mice administered with berbamine [48]. These pathways have also been shown to be regulated by berbamine in triple-negative breast cancer cells [49].

Berbamine increased the intracellular ROS levels via the downregulation of antioxidative genes such as NRF2, SOD2, GPX-1 and HO-1. ROS accumulation led to the activation of the intrinsic apoptotic pathway by an increase in the ratio of Bax/Bcl-2 [50]. A rapidly growing understanding of the complexity within NF-κB signaling pathways has revealed some of the mechanisms that are utilized by the NF-κB family to regulate gene expression during carcinogenesis and metastasis. IKK (an inhibitor of IκB kinase) phosphorylated IκB and relieved inhibitory effects of IκB on NF-κB. Berbamine suppressed p-P65 and p-IκBα levels in bladder cancer cells. Berbamine induced significant shrinkage of the tumors in mice subcutaneously xenografted with T24 cancer cells [51]. 

Berbamine induced the phosphorylation of the non-receptor tyrosine kinase Src in Na^+^/K^+^-ATPase-dependent manner, consequently resulting in the activation of p38-MAPK and EGFR-ERK transduction cascades [50]. Importantly, Na^+^/K^+^-ATPase ligand ouabain also induced EGFR, IGF1R, Src, ERK1/2 and p38-MAPK phosphorylation in HCC cells. Intriguingly, the treatment of HCC cells with EGFR or Src inhibitors blocked berbamine-mediated ERK1/2 phosphorylation. Furthermore, sorafenib inhibited the activation of EGFR, Src, p38-MAPK and ERK1/2 by berbamine and ouabain. Berbamine and sorafenib combinatorially inhibited the growth of tumors in mice xenografted with HepG2 cells [50].

Detoxified pneumolysin derivative ΔA146Ply, a pneumolysin mutant, has been shown to be effective against cancer [52]. ΔA146Ply and berbamine synergistically reduced the growth of tumors in mice transplanted with 4T1 cells. Importantly, the ratio of CD4^−^CD8^−^ T cells was found to be considerably enhanced in the combinatorically treated group, signifying an antitumor role played by CD4^−^CD8^−^ T cells in the combinatorically treated group. Furthermore, the ratio of Treg (regulatory T) cells was reported to be notably suppressed in mice combinatorically treated with ΔA146Ply and berbamine. Pulmonary metastatic lesions were also noted to be reduced in mice combinatorically treated with ΔA146Ply and berbamine [52].

The loss of apoptosis is a critical step that fuels carcinogenesis and metastasis. Researchers are focusing on different strategies to restore apoptosis in resistant cancer cells [53,54,55].

**Figure 3:**
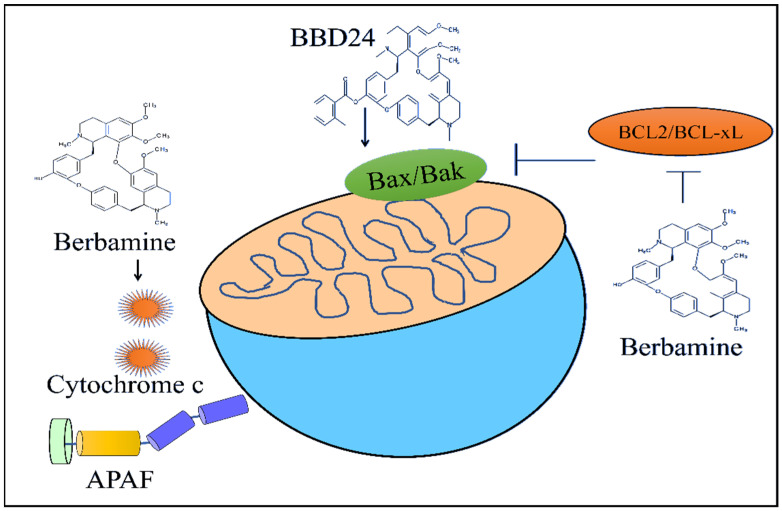
Berbamine reduced Bcl-XL and Bcl-2 but enhanced the release of cytochrome c. 2-methylbenzoyl berbamine (BBD24) increased Bax level.

2-Methylbenzoyl berbamine (BBD24) time- and dose-dependently inhibited the growth of the osteosarcoma HOS and MG63 cells. BBD24 induced an increase in the levels of BAX in HOS cells (Figure 3). Furthermore, cleaved caspase-8, cleaved caspase-9 and cleaved caspase-3 were found to be significantly enhanced in BBD24-treated OS cells. BBD24 and cisplatin induced a regression of the tumors in mice inoculated with HOS cells [56].

Berbamine led to a reduction in the levels of survivin in prostate cancer cells. The berbamine-mediated activation of caspase-9 and caspase-3 was blocked by methazolamide (inhibitor of cytochrome c). Berbamine inhibited tumor growth rates in athymic mice injected subcutaneously with PC-3 cells [57].

Berbamine downregulated the levels of anti-apoptotic proteins Bcl-XL and Bcl-2 in imatinib-resistant BCR-ABL-positive K562 cells (Figure 3). Berbamine induced an increase in the levels of Bax and cytochrome C. Berbamine effectively inhibited leukemia xenograft growth in mice injected subcutaneously with K562-r cells (Figure 3) [58].

Berbamine induced apoptosis in HepG2 cells. It upregulated the expression of Fas in HepG2 cells. Berbamine induced tumor regression in HepG2 xenograft models [59]. Berbamine activated the intrinsic apoptotic pathway in SMMC7721 cancer cells [60]. However, there is a need to drill down deep into the berbamine-mediated apoptotic pathway for a better understanding of the regulation of pro-and anti-apoptotic proteins by berbamine for the induction of apoptosis in therapy-resistant cancer cells.

## 11. Concluding Remarks

Rapidly emerging, cutting-edge research works have provided new horizons for preclinical-to-clinical translatability of molecularly targeted cancer therapies. Based on the sizzling insights gleaned from decades of cutting-edge research, researchers are developing a better understanding of the pharmacological properties of myriad natural products. Overwhelmingly increasing, high-quality, scientifically verified information has enabled interdisciplinary researchers to unravel remarkable medicinal properties of natural products for the treatment of different diseases. Although researchers have reported the promising potential of berbamine as a cancer-chemopreventive agent, berbamine has been shown to pharmacologically target different cellular pathways (shown in Table 1).

There are some major knowledge gaps in our understanding of the regulation of transduction cascades by berbamine. The regulation of non-coding RNAs by berbamine is still inadequate. Identification of the most plausible target miRNAs, lncRNAs and circRNAs will be effective, and rationally designed preclinical studies using oncogenic non-coding RNA inhibitors and tumor suppressor non-coding RNA mimics will be beneficial. Likewise, the use of nanotechnologically delivered berbamine will be effective in targeted therapy in tumor-bearing mice. Although intriguing evidence indicates Berbamine as a good candidate for the adjuvant of canonical cancer therapies, further studies and clinical trials are needed to better understand the effects of this natural compound in humans.For future studies, it is imperative to have more pre-clinical and clinical evaluations on the efficacy and safety of berbamine by integrating novel technologies in precision medicine and pharmacology.

## Figures and Tables

**Table 1:** Pharmacological targets of berbamine.

Berbamine Its Derivates and Combinations	Target Cells	Target Proteins	Effects on Target Proteins	References
Berbamine plus Sorafenib	PRF-PLC-S, HCC-Lm3, HL-7702 (Hepatocellular carcinoma cells)	p-STAT3	Inhibition	[6,50]
Berbamine	U266, RPMI8226, KM3 (Multiple myeloma)	CaMKII p-ERK p-STAT3	Inhibition	[7,11]
Berbamine plus Gefitinib	PANC-1, MIA PaCa-2 (Pancreatic cancer)	p-STAT3	Inhibition	[8,11]
Berbamine plus Radiation	FaDu, KB (HNSCC cells)	p-STAT3 Bax/Bel-2	Inhibition	[10]
4-Chlorobenzoyl berbamine	OCI-Ly3 (DLBCL cell line)	CaMKIl JAK2/STAT3 c-Mye	Inhibition	[22]
4-Chlorobenzoyl berbamine	U266, RPMI 8226, MMI.R and MMI.S (Multiple myeloma)	FOX03a, Bim	Increase	[11]
		p-STAT3, p-AKT	Inhibition	
Berbamine	A549 (lung cancer cells)	P53	Increase	[48,49]
		MDM2	Inhibition	
Berbamine	HepG2 SMMC7721	Fas	Increase	[55,56]
Berbamine	BCR-ABL-positive K562 cells	Bel-XL, Bcl-2	Inhibition	[51,54,56,58]
		Bax, cytochrome	Increase	
2-Methylbenzoyl berbamine	HOS and MG63 (Osteosarcoma cells)	BAX caspase-8, caspase cleaved caspase 3	Increase	[56]

## Data Availability

Not applicable.
